# Generation of transgenic chickens expressing the human erythropoietin (*hEPO*) gene in an oviduct-specific manner: Production of transgenic chicken eggs containing human erythropoietin in egg whites

**DOI:** 10.1371/journal.pone.0194721

**Published:** 2018-05-30

**Authors:** Mo Sun Kwon, Bon Chul Koo, Dohyang Kim, Yu Hwa Nam, Xiang-Shun Cui, Nam-Hyung Kim, Teoan Kim

**Affiliations:** 1 Department of Physiology, Daegu, Republic of Korea; 2 Department of Animal Sciences, Chungbuk National University, Cheongju, Republic of Korea; University of Connecticut, UNITED STATES

## Abstract

The transgenic chicken has been considered as a prospective bioreactor for large-scale production of costly pharmaceutical proteins. In the present study, we report successful generation of transgenic hens that lay eggs containing a high concentration of human erythropoietin (hEPO) in the ovalbumin. Using a feline immunodeficiency virus (FIV)-based pseudotyped lentivirus vector enveloped with G glycoproteins of the vesicular stomatitis virus, the replication-defective vector virus carrying the *hEPO* gene under the control of the chicken ovalbumin promoter was microinjected to the subgerminal cavity of freshly laid chicken eggs (stage X). Stable germline transmission of the *hEPO* transgene to the G_1_ progeny, which were non-mosaic and hemizygous for the *hEPO* gene under the ovalbumin promoter, was confirmed by mating of a G_0_ rooster with non-transgenic hens. Quantitative analysis of hEPO in the egg whites and in the blood samples taken from G_1_ transgenic chickens showed 4,810 ~ 6,600 IU/ml (40.1 ~ 55.0 μg/ml) and almost no detectable concentration, respectively, indicating tightly regulated oviduct-specific expression of the *hEPO* transgene. In terms of biological activity, there was no difference between the recombinant hEPO contained in the transgenic egg white and the commercially available counterpart, *in vitro*. We suggest that these results imply an important step toward efficient production of human cytokines from a transgenic animal bioreactor.

## Introduction

Human erythropoietin (hEPO), a cytokine hormone of renal origin, is required for red blood cell production, and it has been indispensable to patients with anemia caused by chronic renal disease or in cancer patients receiving chemotherapy [1, 2]. The molecular weight of hEPO is 30.4 Kd and consists of 165 amino acids with three *N*-linked (Asn^24^, Asn^38^, and Asn^83^) and one *O*-linked (Ser^126^) carbohydrate chain [3].

At present, most of the human glycosylated therapeutic proteins are produced from an animal cell culture system. However, only a few world class pharmaceutical companies are able to invest tremendous amounts of funds for setting up such a system [4]. As an alternative, the use of secretory glands as bioreactors has been intensively studied over the last three decades. Initially, the mammary gland was a primary candidate for the bioreactor. However, the number of successful trials, in terms of commercialization, was very few [5, 6], mainly due to the long generation time for domestic farm mammals and the difficulties in purifying recombinant therapeutic material from a complex composition of milk. The use of chicken oviducts as bioreactors was thought to circumvent these problems, and has several advantages including a shorter generation time, high fecundity, little expenditure in breeding and maintaining a transgenic chicken flock [7, 8]. In addition, the sterile environment inside the egg shell and high concentration of protease inhibitors in egg whites is believed to afford an ideal condition for preserving the biological activity of exogenous pharmaceutical proteins for long periods [9]. Moreover, compared to milk proteins of mammals, easier purification of recombinant proteins due to less biochemical complexity of the egg white proteins and more similar glycosylation arrangements of some chicken glycoproteins to those of humans has been reported elsewhere [10].

Regarding transgenic chicken-mediated human cytokine production, the generation of some transgenic chickens has been documented so far that express monoclonal antibodies [11, 12], human interferon α-2b [9], human interferon β-1a [12], and human erythropoietin [13, 14, 15, 16]. All of these trials used vectors derived from viruses of the Retroviridae family including the Avian Leukosis Virus (ALV) [9], Murine Stem Cell Virus (MSCV) [11, 13, 14] Moloney Murine Leukemia Virus (MoMLV) [15, 16], and Equine Infectious Anemia Virus (EIAV) [12]. ALV, MSCV and MoMLV belong to the retrovirus genus, while EIAV is in the lentivirus genus [17]. To drive the expression of these cytokine genes, nontissue-specific ubiquitous promoters, such as chicken β-actin [11, 13, 14] the cytomegalovirus (CMV) promoter [9, 16], or the tissue-specific ovalbumin promoter [12] was used.

In this work, we generated transgenic chickens using a feline immunodeficiency virus (FIV)-based lentivirus vector designed to express the *hEPO* gene under the control of the chicken ovalbumin promoter. The resulting transgenic chickens produced biologically functional hEPO as a component of egg whites at one of the highest levels ever reported. In addition, we were able to confirm stable germline transmission of the *hEPO* transgene to the chickens of the next generations.

## Materials and methods

### Construction of viral vectors

**Fig 1:**
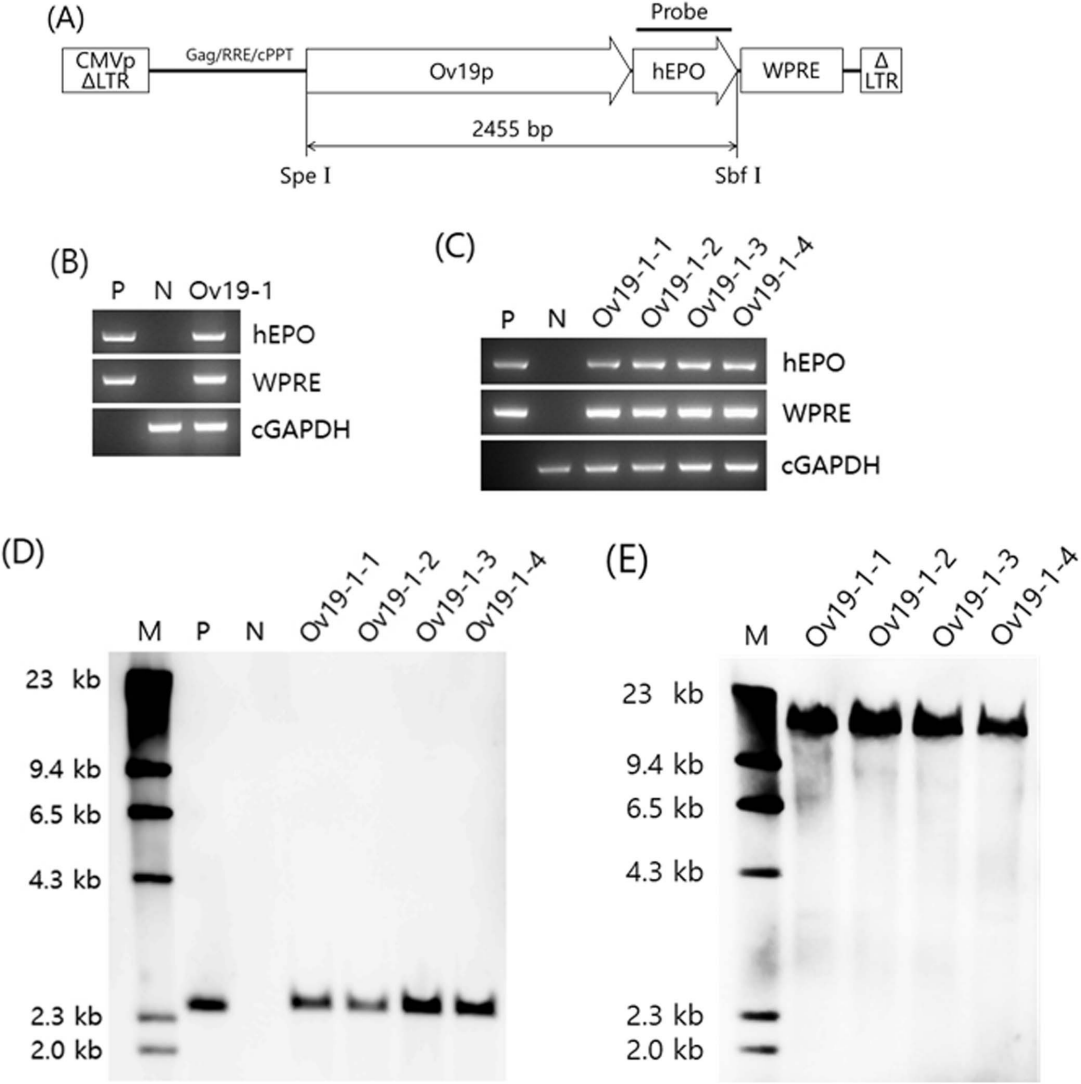
**(A) Structure of the FIV-Ov19-hEPO provirus.** CMV/ΔLTR, a hybrid CMV promoter fused to the FIV long terminal repeat devoid of U3 region; Gag, cis-acting sequence helping efficient packaging of virus transcripts; RRE, coding sequence for the rev response element involved in packaging of the virus particles; cPPT, central polypurine track involved in integration of proviruses into the host cell genome; Ov19p, ovalbumin promoter, hEPO, human erythropoietin gene cDNA; WPRE, woodchuck hepatitis virus posttranscriptional regulatory element sequence; ΔLTR, long terminal repeat with deletion of the U3 region to prevent replication-competent virus production after integration into the host cell genome. The approximate position of the probe for Southern blotting is shown just above the *hEPO* gene. Selected restriction enzyme sites in the provirus sequence are also indicated. In this construct, expression of the *hEPO* gene is driven by the ovalbumin promoter. Drawing is not to scale. **(B & C) PCR analysis of G**_**o**_ **(B) and G**_**1**_ **(C) transgenics.** lane P, plasmid pFIV-Ov19-hEPO; lane N, non-transgenic control chicken, lane Ov19-1, a founder transgenic rooster of G_0_ generation; lanes marked with Ov19-1-1 ~ Ov19-1-4, four transgenic G_1_ generation chickens sired by a transgenic G_0_ rooster. **(D & E) Southern blot analysis of G**_**1**_ **transgenic chickens.** Genomic DNA of chickens was double-digested with *Spe* I and *Sbf* I (D) or single-digested with *Spe* I (E), then hybridized with the hEPO gene probe. Lane M, molecular size markers; the remaining lanes are same as indicated in (B & C).

The plasmid pFIV-Ov19-hPOW (Fig 1A) was constructed by replacing a 1.5-kb internal fragment (from the internal CMV promoter to the Puromycin-resistant gene) of pCDF1-MCS2-EF1-Puro with the sequences of the ovalbumin promoter linked to the *hEPO* gene cDNA. pCDF1-MCS2-EF1-Puro (purchased from System Biosciences, Palo Alto, CA, USA) was derived from the feline immunodeficiency virus (FIV). Like human immunodeficiency virus (HIV), FIV belongs to a lentivirus of the retroviridae family [17], and it has been proven that lentivirus-based vector is much superior to traditional retrovirus-based vector in terms of transducing efficiency especially in non-dividing cells. Similar to HIV causing immunodeficiency in humans, FIV is pandemic and causes immunodeficiency in domestic cats. Compared to the HIV-derived vectors, the vectors of FIV-derived have several biosafety advantages. For example, FIV does not cause human disease, or even cross-react with HIV serologically, etc [18]. The ovalbumin promoter sequence (Genebank accession number AC159826.1) of pFIV-Ov19-hPOW consists of a 676 bp fragment (-3790 ~ -3115) of estrogen response element (ERE) and a 1179 bp fragment (-1132 ~ +47) covering the sequences of the steroid-dependent regulatory element (SDRE), negative regulatory element (NRE) and small upstream region of exon 1. The hEPO cDNA (582 bp) at the 3’ end of the ovalbumin promoter was purchased from Cytokine Bank in Korea. The sequence of the woodchuck hepatitis virus posttranscriptional regulatory element (WPRE) at the 3’ end of the *hEPO* gene was employed to boost the expression of the *hEPO* gene [19].

### Preparation of the retroviral vector virus

Virus-producing cells were constructed by transfecting 293 FT cells. Briefly, 5 x 10^7^ 293FT cells were plated in a 100-mm dish with the mixture consisting of 9 ml of complete cell culture medium plus 1 ml of calcium/phosphate transfection solution prepared with 10 μg each of pFIV-Ov19-hPOW, pVSV-G, and pFIV-34N. The 293FT packaging cells and two kinds of plasmid (pVSV-G, and pFIV-34N) were purchased from System Biosciences (Palo Alto, CA, USA). The transfection medium mixture was replaced with fresh culture medium after 8 hr post-transfection, and then the culture medium containing the viruses packaged with vesicular stomatitis virus G glycoprotein (VSV-G) was collected at 48 hr post-transfection. To prepare the virus solution for microinjection, the medium taken from the virus-producing cells was centrifugally concentrated 1,000-fold and sterilized by filtering through a 0.45-μm pore-size filter [20].

### Production of transgenic chickens

In transferring the foreign gene in the chicken embryos, an *ex ovo* approach requiring donor and recipient eggs was employed [21]. The donors were new-laid eggs corresponding to stage X developmentally [22] and weighing approximately 60 g. For the recipients, we used the eggs weighing approximately 65 g (for the first three days of incubation) or approximately 90 g (for the remaining days). All eggs used in the experiment were obtained from the ISA brown hens. Microinjection of around 2.5 μl of the concentrated virus stock corresponding to approximately 5.0 x 10^6^ Colony Forming Unit (CFU) to the subgerminal cavity of the embryo and incubation of the manipulated eggs until hatching were done following our established protocol [15]. Briefly, all contents including egg white and egg yolk of a donor egg were decanted to an empty recipient eggshell having a drilled round hole of approximately 35-mm in diameter at the blunt end of the egg, and then concentrated virus stock was injected into the subgerminal cavity of the donor egg. To facilitate the virus infection, polybrene (10 μg/ml) was added to the virus stock. After microinjection, filling with additional ovalbumin to the brim of the recipient egg shell was followed by sealing with plastic wrap without leaving an empty space. The manipulated eggs were incubated at 37°C with 60% humidity. Each embryo that underwent 3 days of the first incubation was transferred to a larger empty recipient eggshell drilled to have a 42-mm circular hole, and then the second incubation was carried out for the remaining 18 days.

### Genomic DNA analyses

Purification of genomic DNA from blood and sperm was done using a G-DEX II genomic DNA extraction kit (Intron Biotechnology, Seoul, Korea) and QIAmp DNA Mini kit (Qiagen, Hilden, Germany), respectively. In PCR amplification of the *hEPO* gene, a primer set was devised based on the GenBank data (accession no. NM_000799.2). Use of the devised primer set (upstream; 5’GCTGAACACTGCAGCTTGAATGAG3’; downstream (5’GGAAGAGTTTGCGGAAAGTGTCAG3’) was expected to produce a 340-bp amplified DNA fragment. Detection of the WPRE sequence was done by amplifying a 315-bp DNA fragment using upstream (5’GGATACGCTGCTTTAATGCCTTTG3’) and downstream (5’CGACAACACCACGGAATTGTCAGT3’) primers. As a control, *glyceraldehyde-3-phosphate dehydrogenase (GAPDH)* gene was amplified using a primer pair of upstream (5’TAGTGGTGCAGACTGGGTAGAGCGAA3’) and downstream (5’ TCCTCTGGAGTGGCAAGAGGAGAAAG 3’) sequences to get a 275-bp amplified DNA fragment. Each 20-μl reaction mixture was prepared with 0.1 μg of genomic template DNA, 10 pmol of each primer, and GoTag^®^ Green Master Mix (Promega, Madison, WI, USA). After 5 min of preheating at 94°C, the reaction included 35 cycles of which each consisted of denaturation (at 94°C for 30 sec), annealing (at 57°C for 30 sec), and extension (at 7 for 30 sec). In Southern blotting, genomic DNA (20 mg) digested with restriction enzymes was electrophoresed on a 0.8% agarose gel. The probe (582 bp) to hybridize with the hEPO cDNA was synthesized using the PCR DIG Probe Synthesis kit (Roche, Basel, Switzerland) with the primer pair of 5’ATGGGGGTGCACGAATGTCC3’ (upstream) and 5’TCATCTGTCCCCTGTCCTGCA3’ (downstream). The hybridized DNA fragment was detected using a DIG luminescent detection kit (Roche).

### Quantitative measurement of hEPO

The hEPO concentration was measured by enzyme-linked immunosorbent assays (ELISAs) using the Quantikine IVD Epo kit (R&D Systems, Minneapolis, MN, USA). Serum and egg white samples diluted with ice-cold PBS approximately 100-fold were stirred gently for 20 min at room temperature. All remaining procedures were done following the manufacturer’s manual. Briefly, after further serial dilutions up to 800-fold, 200 μl of each diluted sample in a well of the microplate was added with the same volume of hEPO-HRP conjugate. After an hour of gentle mixing, each well was washed with Wash Buffer 4 times, and then 200 μl of tetramethylbenzidine (TMB) substrate solution was added to each well. Approximately 20 min of incubation in the dark was allowed before adding the stop solution (100 μl per well) and measuring the optical density at 450 nm. Standard curve and concentration of each sample were obtained using standard samples of various hEPO concentration provided by the Quantikine IVD Epo kit manufacturer) and operating iMark^Tm^ microplate reader (Bio-Rad, Hercules, CA, USA).

### Measurement of the biological activity of hEPO *in vitro*

The biological activity of hEPO in the egg white of the transgenic eggs was determined by exploiting hEPO-mediated inducible proliferation of TF-1 human erythroleukemia cells (ATCC No. CRL-2003) *in vitro* [23]. Briefly, approximately 1.0 × 10^4^ TF-1 cells cultured in RPMI 1640 medium supplemented with 10% FCS and 2 ng/ml of human Granulocyte–Macrophage Colony-Stimulating Factor (hGM-CSF; purchased from Komabiotech, Korea) were seeded in each well of a 96-well microtiter plate with 50 μl of RPMI 1640 supplemented with 2% FBS. The same volume of each sample after two-fold serial dilutions was added to the wells in triplicate before incubation at 37°C in a humidified 5% CO_2_ atmosphere for 48 h. After adding 10 μl of 3-[4,5-dimethylthiazol-2-yl]-2,5-dimethyltetrazolium bromide (MTT) to each well, cells were incubated at 37°C for 4 h in the same atmospheric condition, and 100 μl of solubilization solution was then added to each well. The samples were incubated again for 16 h at 37°C in a same humidified 5% CO_2_ atmosphere before measuring the optical density of each sample at 595 nm. The Cell Proliferation Kit I, consisting of MTT and the solubilization solution, was purchased from Roche (Germany). The hEPO used for the standard was purchased from NIBSC (Potters Bar, Hertfordshire, UK).

### Glycosylation assay

Denaturation of the protein by preheating the sample in denaturing buffer at 100°C for 10 min was carried out. To remove *N*-linked carbohydrates, each 20 μl of the reaction mixture was prepared with 20 ng of hEPO, 500 U of PNGase F, 2 μl of 10X GlycoBuffer 2, and 10% NP 40. To remove the *O*-linked carbohydrates, the samples were treated with 40,000 U of *O*-glycosidase and 50 U of neuraminidase. To take out both *N*-linked and *O*-linked carbohydrates, the same amount of hEPO sample was treated with 500 U of PNGase F, 40,000 U of *O*-glycosidase and 50 U of neuraminidase. All materials including enzymes and buffers were purchased from New England Biolabs (Ipswich, MA, USA). After incubation at 37°C for 4 hours, the samples underwent electrophoresis were transferred to a nitrocellulose membrane. After 1 h of blocking in TBS with 0.03% Tween-20 (MTBST) and 5% skim milk, the membrane was incubated with a mouse monoclonal anti-hEPO (R&D Systems, Minneapolis, USA) in MTBST (1/5,000 dilution) for 16 h. Following washing with TBST alone three times, the membrane was incubated with HRP-conjugated rabbit anti-mouse IgG (Abcam, Cambridge, UK) in MTBST (1/15,000 dilution) for 1 h. After washing three times with TBST, the detection of chemiluminescence was done by adding West Dura Extended Duration substrate (Thermo Fisher Scientific, MA, USA).

### Animal ethics statement

All animal experimental procedures in this work were performed under the guidelines of the Ethics Committee for Experimental Animals of the Catholic University of Daegu School of Medicine, Korea (permission # 2012-0216-CU-AEC-04-Y).

## Results

### Generation of hEPO transgenic chickens

We injected concentrated recombinant FIV-based lentivirus vector stock directly into the blastoderm of chicken embryos to produce founder transgenic chickens corresponding to the G_0_ generation. Of 208 eggs injected with FIV-Ov19-hEPOW, 10 chicks (or 4.8% of the total eggs manipulated) hatched, and all of them (eight females and two males) were transgenic. One of the two male chicks was identified to carry the transgene in the genome of sperm cells (Fig 1B). The PCR positive transgenic G_0_ rooster (ID # Ov19-1) was mated to wild-type hens to produce 518 G_1_ progeny. PCR analysis of blood samples taken from these progeny identified four female G_1_ transgenic offspring (Fig 1C), corresponding to a 0.77% germline transmission rate. In subsequent Southern blot analysis, digestion of DNA samples with *Spe I* and *Sbf I* followed by hybridization with a probe specific to the *hEPO* gene detected an expected 2.45 kb band corresponding the combined size of the ovalbumin promoter and *hEPO* gene (Fig 1D). This indicated stable genomic integration of the unimpaired transgene in all four G_1_ transgenics. Blotting of DNA samples digested with Spe I, which makes only one cut within the provirus (Fig 1A), resulted in a single band in all samples from four G1 transgenic chickens (Fig 1E). This result suggested that only one insertion of the provirus occurred in the genome of all four G1 birds. Interestingly, the integration of the transgene seemed to occur at the same site of the chromosome because the MW of each band was seemingly the same (Fig 1E).

### Production of hEPO in transgenic chickens

**Fig 2:**
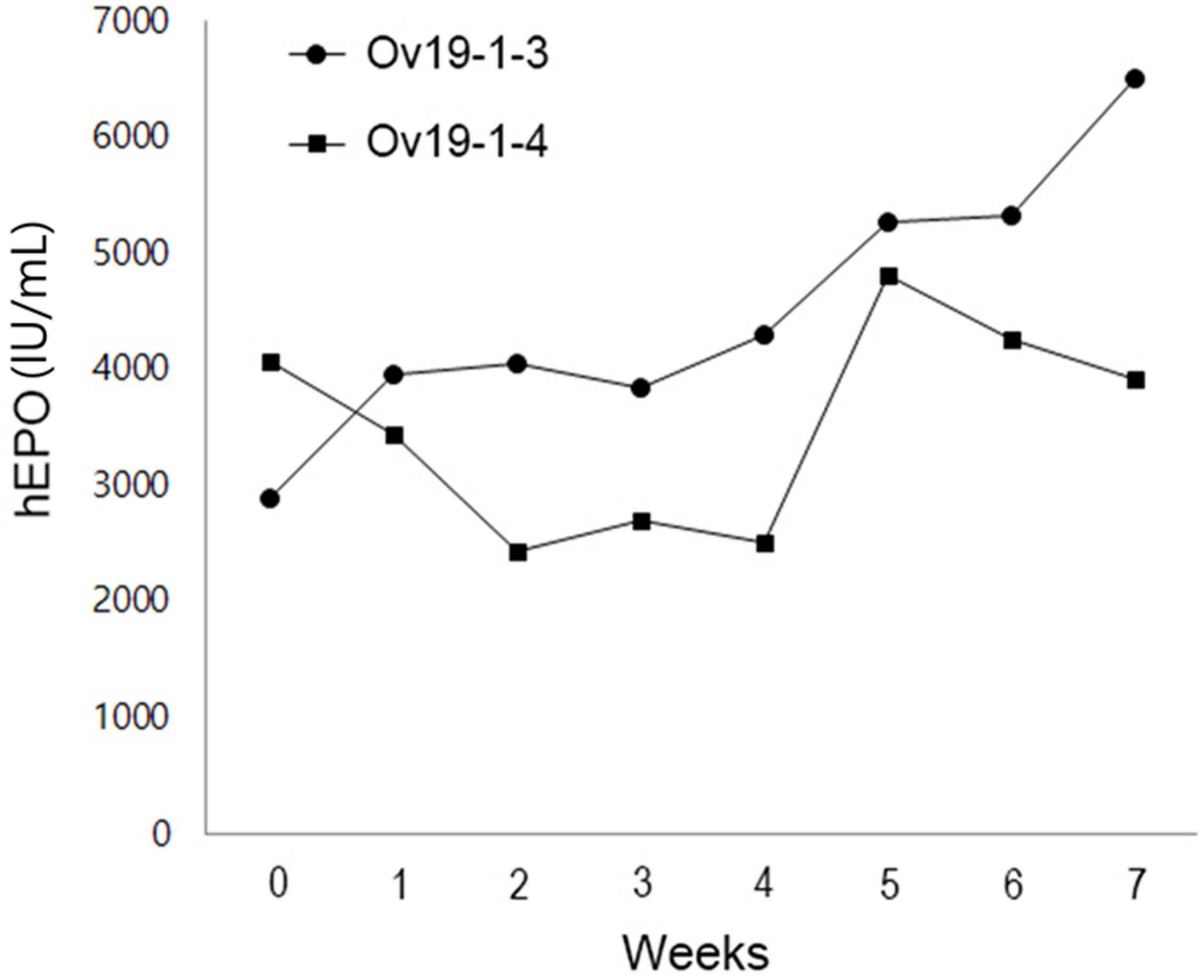
Expression of the *hEPO* gene in two G_1_ transgenic hens (#Ov19-1-3 and #Ov19-1-4). The levels of hEPO in the egg white were measured with an ELISA. The first egg for hEPO analysis was collected from a hen that was 25 weeks of age (marked ‘0’ on ‘X’ axis). Measurement was done once a week for the period of seven weeks.

Among four chickens of the G_1_ generation produced by using FIV-Ov19-hPOW, two were dead before puberty for unknown reasons. Therefore, only two hens were available for hEPO analysis. Quantitative measurement of the hEPO concentration in the blood using an ELISA showed an 18 and 25 mIU/ml (0.15 ~ 0.2 ng/ml) hEPO concentration (S1 Fig). In the case of two egg white samples, the highest hEPO concentration was 4,810 IU/ml (or 40.1 μg/ml) for Ov19-1-4 hen or 6,600 IU/ml (or 55.0 μg/ml) for Ov19-1-3 hen (Fig 2). This result clearly indicated tight regulation of oviduct-specific expression of the *hEPO* transgene, confirming our expectation.

### Analyses of recombinant hEPO proteins

**Fig 3:**
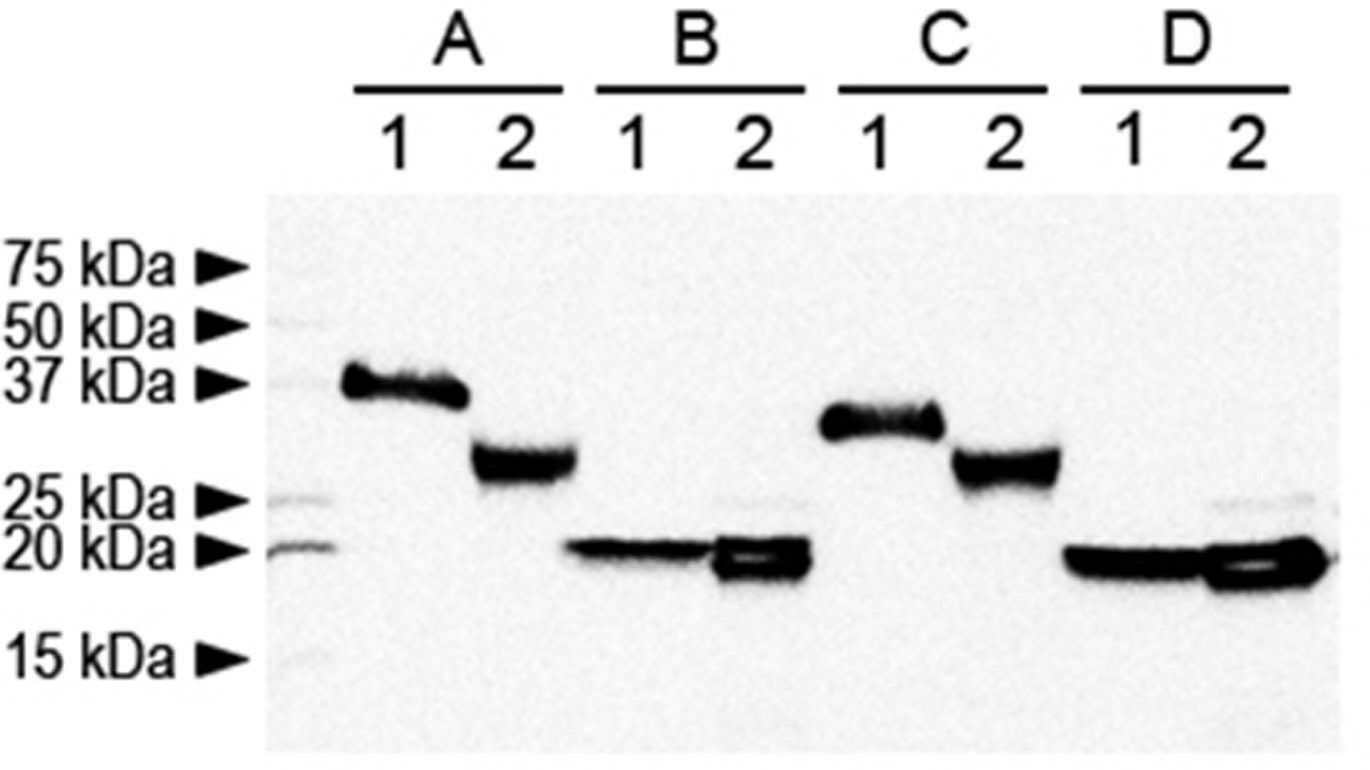
Analysis of the glycosylation pattern of egg white-hEPO derived from transgenic eggs. **(A)** Intact samples treated with no enzyme. **(B)** Samples treated with PNGase F to remove *N*-linked carbohydrates. **(C)** Samples treated with *O*-glycosidase and neuraminidase to remove *O*-linked carbohydrates. **(D)** Samples treated with a combination of PNGase F, *O*-glycosidase and neuraminidase to remove all carbohydrate chains. ‘1’ and ‘2’ under each uppercase alphabetical letter indicate ‘CHO cell-derived control hEPO’ and ‘egg white-hEPO’, respectively.

Recombinant hEPO in the eggs produced from one G_1_ transgenic hen (ID # Ov19-1-3) was analyzed by Western blotting. Unexpectedly, as shown in Fig 3A, the molecular weight of the recombinant hEPO protein in the egg white was smaller than that of the CHO cell-derived control. We suspected that a lesser degree of glycosylation compared to the control might be the main cause and so we performed enzymatic digestion analyses. Digestion of the hEPO samples with PNGase F, which is known to cleave the *N*-linked carbohydrates, resulted in a decreased molecular size profile in both control and egg white EPO samples (Fig 3B) compared with the band sizes of two samples untreated with PNGase (Fig 3A). This indicated the presence of *N*-linked carbohydrates in the recombinant hEPO derived from both CHO cell control and transgenic egg white. Another digestion with both *O*-glycosidase and neuraminidase to remove the *O*-linked sugar moiety also resulted in a slightly decreased molecular profile in both control and egg white EPO samples (Fig 3C) compared with the band sizes of two samples untreated with two enzymes (Fig 3A), indicating the presence of *O*-linked glycosylation in both hEPO derived from the CHO cell and egg whites. However, the smaller molecular weight of the egg white-derived hEPO compared to the CHO cell-derived control (Fig 3C) suggested that the degree of *N*-linked glycosylation of egg white hEPO was less than that of control hEPO. Further analysis was done with the recombinant hEPO that underwent complete removal of all sugar residues. Triple digestions of samples with PNGase, *O*-glycosidase and neuraminidase resulted in the same molecular weight of hEPO regardless of the source (Fig 3D). This confirms that it is not protein itself but the glycosylation pattern that causes different molecular weights between CHO cell derived hEPO and egg white-derived hEPO, as shown in Fig 3A.

**Fig 4:**
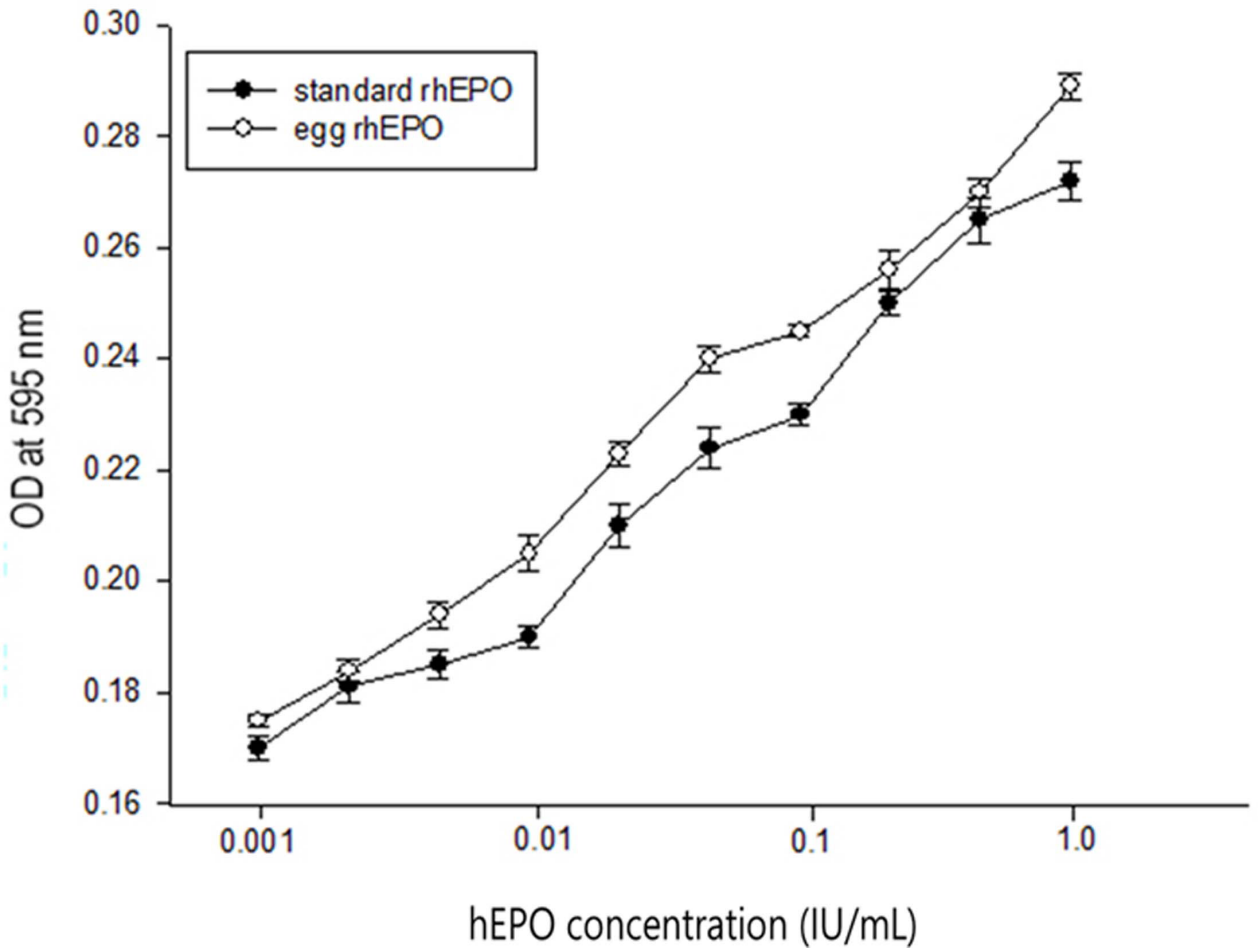
The biological activity of egg white-hEPO. The proliferation of TF-1 cells cultured in the presence of various concentrations of hEPO derived from the eggs laid by a transgenic hen (chicken ID #Ov19-1-3, open circle,) was evaluated as described in the ‘‘Materials and Methods” section. Compared with a commercially available recombinant (closed circle), no significant difference in biological activity was determined by analysis of variance using the general linear model (GLM) procedure in the Statistical Analysis System (SAS).

Finally, we evaluated the *in vit*ro biological activity of the transgenic protein over a 10^−3^ ~ 1 U/ml concentration range, for which the proliferation rate of TF-1 cells was proportional to the hEPO concentration. Interestingly, despite a lower degree of glycosylation, hEPO from the transgenic chicken egg white was as active as the recombinant hEPO derived from CHO cells (p>0.05) (Fig 4).

## Discussion

In the study of transgenic animal generation, choosing an appropriate vector and promoter has been a matter of foremost consideration. Although there has never been any transgenic chicken reported as being used in a study so far, the reason for choosing a FIV-based lentivirus vector in this work was to minimize inherent bio-hazard problems of conventional retrovirus- or lentivirus-derived vectors. In addition, proven results in our previous work with transgenic animals encouraged us to use a FIV-based lentivirus vector in this study [24]. Regarding the promoter, by using a tetracycline inducible promoter [15] or non-tissue-specific ubiquitous cytomegalovirus (CMV) promoter [16], we previously generated transgenic chickens expressing the *hEPO* gene under the control of either the tetracycline-inducible promoter or ubiquitous CMV promoter. In the case of a tetracycline-inducible promoter, we were able to demonstrate inducible expression of the *hEPO* gene, as much as a 720-fold increase, just by providing the formula feed mixed with tetracycline. However, the overall expression level of the *hEPO* transgene was far lower than expected. The hEPO concentration in both serum and egg whites was no more than 360 IU/ml (≒3 μg/ml) [15]. We inferred that low expression of the transgene was due to the weak strength of the tetracycline-inducible promoter. When we used the CMV promoter to drive the *hEPO* gene, we could get the chickens producing a high level of hEPO in the blood (90 ug/ml in blood) [16]. The chickens, however, showed various detrimental physiological disturbances presumably due to non-tissue specific and uncontrolled constitutive expression of the transgene.

To solve these problems found in the previous studies, we used an ovalbumin promoter for oviduct-specific expression of the *hEPO* gene in the transgenic chickens of this work. We reasoned that high expression of the *hEPO* gene in the confined organ such as oviduct might maximize the efficiency of hEPO production with minimal various physiological disturbances. In this study, using the FIV-Ov19-hEPOW vector carrying the size of 1.9 kb ovalbumin promoter, we were able to get transgenic chickens producing hEPO mainly in the egg white as much as 4,810 ~ 6,600 IU/ml (40.1 ~ 55.0 μg/ml). As expected, almost no expression of the *hEPO* gene was detected in blood, indicating successful oviduct-specific expression of the transgene. Consequently, unlikely our previous transgenic chickens ubiquitously expressing the *hEPO* gene in uncontrollable manner [16], no significant physiological abnormalities were observed. Given that the volume of egg white in an egg is 30 ml, each egg produced from our transgenic hens was estimated to contain functional hEPO as much as 1.2 ~ 1.65 mg. At present we are beginning to get G_2_ generation chicks from a hemizygous G_1_ transgenic hen (chicken ID #Ov19-1-3) mated to a wild type non-transgenic rooster. PCR analysis identified six out of eleven G_2_ offspring was transgenic, indicating the germ transmission ratio from G_1_ to G_2_ (6/11 = 54.5%) was close to Medelian ratio (S2 Fig). Unexpectedly, however, the hen laid only 3 eggs per week on average. As indicated in the ‘Materials and method’, all eggs used in this study were obtained from the ISA brown hens, each of which was supposed to lay approximately 6 eggs a week. Because too many factors are involved in egg laying and the number of G_1_ hens used to collect eggs was limited, it is inappropriate to draw any firm conclusion about influence of the *hEPO* transgene on the low egg-laying rate.

In the assay of glycosylation, which has been known to be critical for appropriate biological activity, we found that the degree of *N*-linked glycosylation of egg white hEPO was less than that of the CHO cell-derived control. A similar phenomenon was also reported elsewhere [13].

Although it was encouraging that the biological activity of hEPO from transgenic chicken egg whites was comparable to the commercially available counterpart *in vitro*, investigation on the less degree of glycosylation needs to be done in future study because less degree of glycosylation might cause a shorter half-life in serum and lesser biological activity *in vivo* [13].

In conclusion, this work is significant because it is the first successful report on the FIV-vector-mediated generation of transgenic chicken that can produce functional hEPO mainly in egg whites. Due to the stable germline transmission of the *hEPO* transgene to the next generations, successful establishment of a new line of transgenic chickens characterized by one of the highest records of functional hEPO production through egg whites might suggest a bright future for the studies of transgenic chicken-mediated production of valuable pharmaceuticals.

## Supporting information

S1 Fig Expression of the *hEPO* gene in G_1_ transgenic chickens. (DOCX)

S2 Fig PCR analysis of 11 G_2_ chickens. (DOCX)
