# Supplementary material for: Generation of transgenic chickens expressing the human erythropoietin (hEPO) gene in an oviduct-specific manner: Production of transgenic chicken eggs containing human erythropoietin in egg whites
Source: PLoS One. 2018 May 30;13(5):e0194721. doi: 10.1371/journal.pone.0194721 (PMC5976184; doi:10.1371/journal.pone.0194721)
Supplement: S1 Fig — (DOCX) [file pone.0194721.s001.docx]

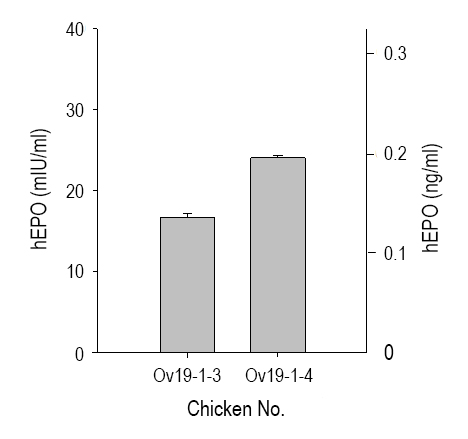


**S1 Fig. Expression of the *hEPO* gene in G_1_ transgenic chickens.**

The levels of hEPO secreted into the blood taken from 25 week-old chicks were measured by ELISA as described in the materials and methods. The data were obtained from experiments performed in triplicate and are presented as the mean±SD.
