# Supplementary material for: Generation of transgenic chickens expressing the human erythropoietin (hEPO) gene in an oviduct-specific manner: Production of transgenic chicken eggs containing human erythropoietin in egg whites
Source: PLoS One. 2018 May 30;13(5):e0194721. doi: 10.1371/journal.pone.0194721 (PMC5976184; doi:10.1371/journal.pone.0194721)
Supplement: S2 Fig — (DOCX) [file pone.0194721.s002.docx]

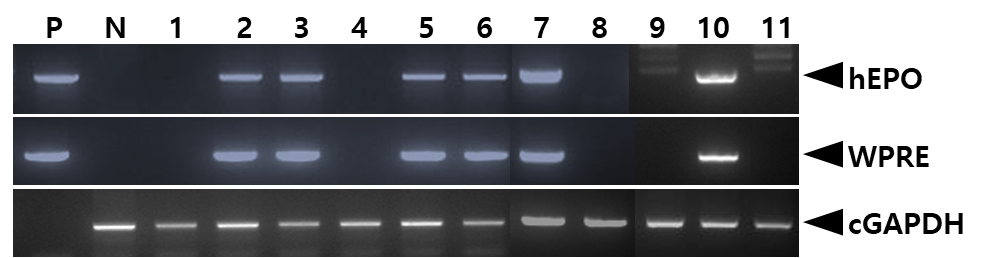


**S2 Fig. PCR analysis of 11 G_2_ chickens.**

**lane P**, plasmid pFIV-Ov19-hEPO; **lane N**, non-transgenic control chicken, **lanes of 1~11**, eleven progeny chickens of G_2_ generation; **hEPO**, human erythropoietin, **WPRE**, woodchuck posttranscriptional response element; **cGAPDH**, chicken glyceraldehydes 3- phosphate dehydrogenase
